# Recent progress on kinetic control of chemical vapor deposition growth of high-quality wafer-scale transition metal dichalcogenides

**DOI:** 10.1039/d1na00171j

**Published:** 2021-05-05

**Authors:** Qun Wang, Run Shi, Yaxuan Zhao, Runqing Huang, Zixu Wang, Abbas Amini, Chun Cheng

**Affiliations:** Department of Materials Science and Engineering, Southern University of Science and Technology Shenzhen 518055 People's Republic of China chengc@sustech.edu.cn 11930238@mail.sustech.edu.cn shir@mail.sustech.edu.cn 11812334@mail.sustech.edu.cn 11812333@mail.sustech.edu.cn 11812324@mail.sustech.edu.cn; Department of Physics and Center for Quantum Materials, Hong Kong University of Science and Technology Hong Kong People's Republic of China; Center for Infrastructure Engineering, Western Sydney University Kingswood NSW 2751 Australia abbasust@gmail.com

## Abstract

2D transition metal dichalcogenides (TMDs) have attracted significant attention due to their unique physical properties. Chemical vapor deposition (CVD) is generally a promising method to prepare ideal TMD films with high uniformity, large domain size, good single-crystallinity, *etc.*, at wafer-scale for commercial uses. However, the CVD-grown TMD samples often suffer from poor quality due to the improper control of reaction kinetics and lack of understanding about the phenomenon. In this review, we focus on several key challenges in the controllable CVD fabrication of high-quality wafer-scale TMD films and highlight the importance of the control of precursor concentration, nucleation density, and oriented growth. The remaining difficulties in the field and prospective directions of the related topics are further summarized.

## Abstract 2

Wafer-scale TMD films are fabricated *via* CVD method, controlling precursor concentration, nucleation density and orientated growth. Precursor concentration is important for the ideal film. Nucleation and orientated growth allow large domain size or single crystalline TMDs.

## Graphical abstract

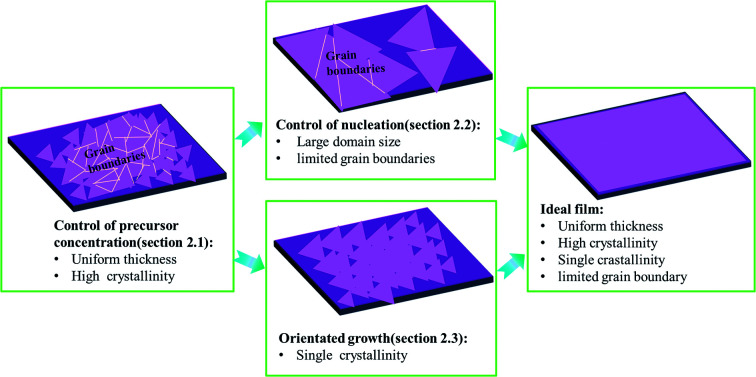

## 1 Introduction

2D transition metal dichalcogenides (TMDs), such as (Mo, W)(S, Se, Te)_2_, have attracted significant attention due to their excellent electrical switching performance,^1–4^ high exciton binding energy,^5–8^ strong optical absorption,^9–11^ and so on. Based on these properties, TMDs have found extensive applications in electronics and optoelectronics, such as field-effect transistors (FETs),^12–16^ photodetectors,^17–19^ and solar cells.^20–22^ For example, MoS_2_-based FETs have exhibited excellent electrical performance with high on/off current ratios of ∼10^8^ and a carrier mobility of 200 cm^2^ V^−1^ s^−1^ at room temperature (RT);^23^ with these characteristics, they have great potential for future electrical circuits that require low stand-by power.

To meet the requirements of modern applications, the controllable fabrication of wafer-scale TMD films with desired properties, such as large domain size, single crystallinity, and good uniformity, is highly required. Compared to commonly used mechanical exfoliation,^23^ chemical vapor deposition (CVD) is believed to be a promising methodology to produce satisfactory TMD materials, especially for uniform thin films at the wafer-scale. However, the CVD growth of TMD materials is very sensitive to the reaction parameters, such as precursors, temperature, gas flow rate, and substrate.^24–26^ Therefore, any small perturbation may largely influence the reaction kinetics and quality of final products, leading to poor reproducibility of reactions. Therefore, it is essential to deeply understand the thermodynamic and kinetic factors involved in the CVD growth of TMDs.

Up to now, the as grown wafer-scale TMD films have always suffered from poor uniformity, high-concentration grain boundaries. Therefore, it remains a big challenge to fabricate wafer-scale TMD films with uniformity and large domain size.^19,24,27^ The uncontrollable grain boundaries or defects on TMD films will largely suppress the carriers passing through the film and thus degrade the device mobility which harms the application of optoelectronic and electronic devices such as field-effect transistors. Therefore, the controllable fabrication of wafer-scale TMDs with uniformity and large domain size has obvious significance for future device application.

Despite recent reviews that have discussed the strategies related to modulating the CVD growth of TMD films,^28–31^ a focused discussion is yet required on the kinetics control of the CVD fabrication of TMDs. Considering growth techniques of tungsten and molybdenum dichalcogenides ((Mo, W)(S, Se)_2_) have been well developed over the past 10 years and thousands of related articles have been published during this period, here, we present a review on the recent advances of controllable CVD growth of wafer-scale, high-quality TMD (mainly focusing on (Mo, W)(S, Se)_2_) thin films *via* precise kinetics control. The efforts made for improving the uniformity and single crystallinity of TMD films are also discussed. Notably, several key challenges are presented for the controllable growth of TMDs accompanied by possible solutions. We also wish the discussion in this review can cause some useful ideas for future research of other interesting TMDs.

## 2 Strategies for controllable fabrication of wafer-scale high-quality TMD films

**Fig. 1:**
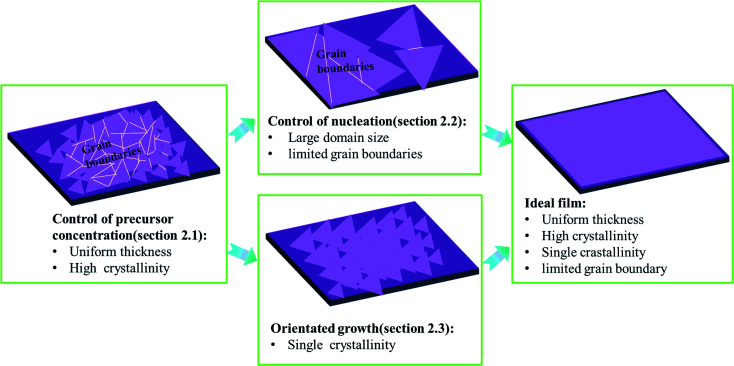
A diagram of the logic structure of Section 2.

As shown in Fig. 1, ideal wafer-scale high-quality TMD films should contain the following features: (1) uniform thickness; (2) high crystallinity; (3) single crystallinity; (4) defect free and limited grain boundaries. However, the CVD growth of TMDs often suffers from a lack of control of the precursor vapor concentrations, leading to uncontrollable reaction kinetics and undesired products, especially with poor crystallinity and uniformity in thickness.^15,19^ Apart from crystallinity and uniformity, it also remains a challenge to obtain large domain size or single crystalline TMDs. Usually, small domain size in TMD film results in a large number of grain boundaries and defects which harm the application of the electronic device.^32^ Therefore, two key issues that are highly desired to solve are: (1) how to control the precursor concentration effectively during the CVD growth of TMDs and (2) how to obtain high-quality TMDs with large domain size and limited grain boundaries. Therefore, in Subsection 2.1, we mainly discuss the control of uniformity and quality of as-grown samples by finely adjusting the precursor concentration. Besides, we highlight that the control of the precursor concentration (Section 2.1) is the prerequisite for the fabrication of wafer-scale TMD films with large domain size (as shown in Fig. 1). Moreover, to enlarge the domain size of TMD film or eliminate grain boundaries, some further strategies can be utilized to suppress the nucleation density or guide the orientated growth of TMD domains, which are discussed in detail in Sections 2.2 and 2.3, respectively.

### 2.1 Control of precursor vapor concentrations

It is worth mentioning that the kinetics control is always the most important topic of CVD reactions as it directly affects the morphology, domain size, and quality of as-grown TMD crystals.^19^ Results of research studies about the CVD growth of TMDs often suffer from a lack of control of the precursor vapor concentrations, leading to uncontrollable reaction kinetics and undesired products. In this subsection, we mainly take a Mo source and a S source as an example of a metal precursor and a chalcogen precursor, respectively, and focus on effective strategies of controlling precursor concentration and mainly focus on the following target precursor with respect to four aspects: (1) solid metal precursor, (2) gaseous metal precursor, (3) liquid metal precursor and (4) chalcogen precursor.

**Fig. 2:**
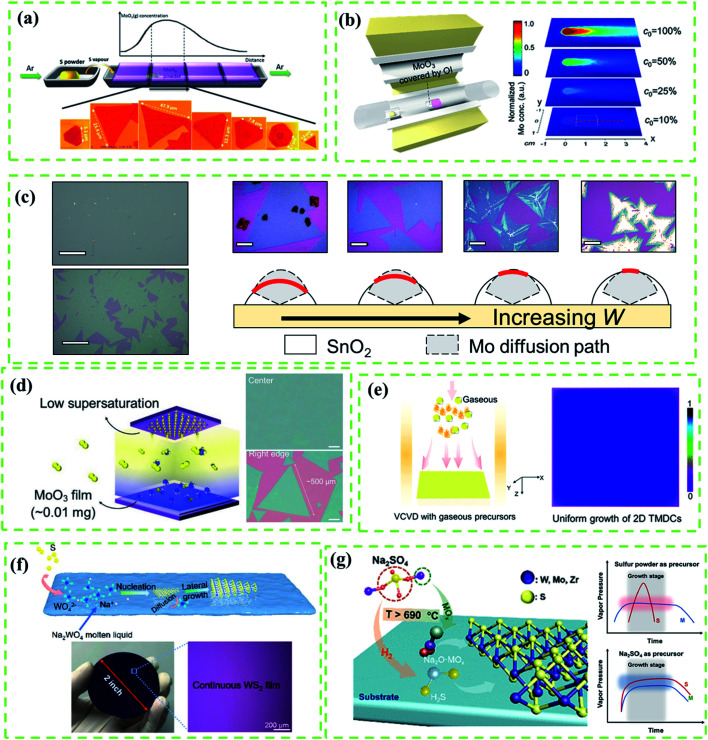
(a) Schematic diagram of the point source supply system for the CVD growth of MoO_3_. MoO_3_ vapor concentrations as a function of the distance between Mo source and substrate results in various sizes and shapes of MoS_2_. Adapted with permission from ref. 33, copyright 2014 American Chemical Society. (b) Schematic diagram of the point source supply system of the OI-assisted CVD growth of MoX_2_ (left) and simulated distribution of the Mo vapor concentration on the substrate with the decreasing concentration of Mo vapor released from the precursor (right) by COMSOL Multi-Physics software using the experimental parameters. (c) Optical images of selected points from the sample (scale bars: 100 μm) and sketch map (right) of the working mechanism of OI in the OIAG reaction (bottom) and optical images (top, scale bars: 20 μm) of MoS_2_ samples prepared under different dosages of SnO_2_ inhibitor (*W* = 4, 5, 6 and 7 mg). (b and c) Adapted with permission from ref. 34, copyright 2020 American Chemical Society. (d) Enlarged diagram of the face source (left) of the CVD growth system and typical optical images of MoS_2_ crystals (right) grown at the center and the right edge of the substrate. Adapted with permission from ref. 19, copyright 2017 Wiley-VCH. (e) Schematics of typical vertical chemical vapor deposition (VCVD) systems using gaseous precursors (left) and as-grown sample with a uniform distribution (right). Adapted with permission from ref. 35, copyright 2020 American Chemical Society. (f) Mechanism illustration of the molten liquid intermediate growth (top); photograph and optical image (bottom) of a 2 inch monolayer WS_2_ film grown on the SiO_2_/Si substrate. Adapted with permission from ref. 36, copyright 2020 American Chemical Society. (g) Schematics for the growth of monolayer TMDs with Na_2_SO_4_ as a sulfur precursor (left), and illustration of the vapor pressure variation of sources with time by using Na_2_SO_4_ as a sulfur precursor (top) and a typical traditional sulfur powder-based method (bottom). Adapted with permission from ref. 37, copyright 2020 American Chemical Society.

As shown in Fig. 2a, Wang *et al.*^33^ reported a typical point source supply system to synthesize MoS_2_ based on the CVD method. They found that the metal vapor concentration was highly determined by the distance between the substrate and metal source while the S vapor concentration was considered as a constant, resulting in the large spatial gradient of the S : Mo ratio in the reaction as well as products with complicated composition. Therefore, fine control of the metal vapor concentration was an inevitable issue for controllable CVD reactions. It was also concluded that an appropriate S : Mo ratio was the prerequisite for the successful fabrication of MoS_2_.

In the CVD fabrication of Mo(S, Se, Te)_2_, MoO_3_ is a typical Mo source under high vapor pressure at 650–800 °C. However, the uncontrollable Mo evaporation often leads to precursor poisoning or contamination of products.^38^ In this regard, precise control of Mo release is considered as an effective method for kinetics control. Nickel oxide (NiO) foam was first used as a barrier to control the deposition rate of Mo precursors on the substrate for the uniform growth of MoS_2_ films, but the evaporation rate was fast which led to uncontrollable reaction kinetics.^15,39^ Moreover, the accelerated Mo release driven by the initial S evaporation, which prohibits the effective kinetics modulation, is yet to be addressed. To solve these problems, Shi *et al.*^34^ provided an oxide-inhibitor-assisted growth strategy to produce high-quality 2D Mo(S, Se, Te)_2_ monolayer films (Fig. 2b) by covering MoO_3_ with an oxide-inhibitor layer (*e.g.*, SnO_2_, TiO_2_, or Al_2_O_3_). The Mo release was suppressed by the oxide-inhibitor layer, reducing the vapor concentration gradient (Fig. 2b), and achieving the effective control of the S : Mo ratio during the reaction process. As shown in Fig. 2c, different dosages of SnO_2_ result in different X : Mo ratios. A small X : Mo ratio facilitates the growth of single-layer MoX_2_, while MoX_2_ multilayers are driven by the high X : Mo ratio. However, the vapor concentration gradient from the point source supply system cannot be thoroughly eliminated; this damages the uniformity of as-grown TMD films (Fig. 2c). To create uniform Mo vapor in the CVD reaction, Lee *et al.*^19^ used a plane source, a thin MoO_3_ film deposited on a Si/SiO_2_ substrate (face source supply setup, Fig. 2d), to produce a highly-crystalline MoS_2_ film with a uniform thickness and large domain size (up to 500 μm) as shown in Fig. 2d. Compared to the point source supply system, the face source supply system can effectively reduce the precursor concentration gradient that is diffused onto the substrate.

To date, high-melting-point metal precursors, such as metal oxides and metal films, have been widely used for the CVD fabrication of various TMD crystals.^3,13,33,34^ However, the complicated evaporation process of these precursors, which is usually assisted by chalcogen sources or hydrogen, makes it difficult to precisely modify the reaction route by adjusting a single variant. By contrast, low-melting-point gaseous precursors, such as MoCl_5_ and WCl_6_, should be ideal choices for effective modulation of reaction kinetics. For instance, Tang *et al.*^35^ prepared polycrystalline monolayer TMD films with centimeter-scale and superb uniformity by using the VCVD system (Fig. 2e) and gaseous precursors (such as MoCl_5_). However, due to uncontrollable nucleation density, the grain size was relatively small (the average domain size of WS_2_ is 9.7 μm). Besides, metal halides (MoCl_5_, WCl_6_, *etc.*) and metal hexacarbonyls (Mo(CO)_6_, *etc.*) inevitably release highly corrosive HCl or carbonaceous residues after decomposition during the CVD reaction, which eventually etch and contaminate products.^40^

Compared with gaseous precursors, some soluble solid transition metal salts such as sodium tungstate (Na_2_WO_4_),^36,41^ sodium molybdate (Na_2_MoO_4_)^42^ and ammonium tungstate ((NH_4_)_2_WO_4_)^43^ are considered as more environmentally friendly and low-cost metal precursors. Besides, these solid transition metal salts can dissolve in water and then can be conveniently spin-coated on a substrate with uniform distribution. An annealing process at high temperature is needed to form the molten liquid precursor during the reaction,^38–40^ which can be treated as a special liquid precursor. For example, Liu *et al.*^41^ introduced a molten-liquid intermediated CVD process for the growth of large-area TMDs with uniform thickness and a continuous monolayer (Fig. 2f). Before the reaction, an annealing process is used to promote the solid precursor to fully transfer to the molten liquid phase which leads to lateral epitaxial growth. However, the nucleation density can't be effectively controlled because the liquid precursor can easily undergo nucleation which thus results in small grain size.

In addition to the control of metal sources, the modulation of chalcogen sources (S, Se, Te) is another important issue that has not been systematically studied. Here we take the control of the S source as an example. Although the morphology, thickness, and domain size of CVD-grown 2D transition metal sulfide films can be affected by the S amount, the experimental outputs often suffer from a lack of reproducibility due to the poor control of S evaporation.^16^ By contrast, gaseous hydrogen sulfide (H_2_S)^4,44^ has been discovered as an effective S source for the growth of transition metal sulfides, but its high toxicity is a major issue. Jin *et al.*^37^ introduced sodium sulfate (Na_2_SO_4_) as a sulfur precursor to effectively control the diffusion of source precursors and balance their mass flux (Fig. 2g). As shown in Fig. 2g (right), the eliminations of Na_2_SO_4_ and metal sources are synchronously released with metal precursor and spanning the entire growth stage, which is difficult to achieve in traditional CVD reactions utilizing the precursor S. However, the addition of Na_2_SO_4_ could inevitably lead to the doping of the Na element that may downgrade the quality of film. Therefore, appropriate chalcogen sources for the controllable fabrication of TMD films are still missing.

In summary, the effective kinetics control during the CVD growth of TMDs requires a precise modulation of precursor concentrations assisted by utilizing appropriate precursors and well-designed reaction systems. According to the experiment results above, the ratio of precursor concentrations plays an essential role in the controllable growth of TMDs. However, it is still unclear how this ratio affects the reaction processes and its working mechanism, due to a lack of evident experimental evidence. The most difficult point is that one can hardly have quantitative monitoring and modulation of the gaseous precursors during the reaction wherein any perturbation can be partly compensated by the self-adjustment of the complicated reaction systems. Perhaps, the *in situ* monitoring of the reaction process can help us acquire useful information and an optimized growth strategy.

### 2.2 Control of nucleation

Monolayer TMD flakes with large single-crystalline domain size that contain limited defects or grain boundaries are highly desired for the devices in practice. However, the controllable growth of large domain size TMD films still remains challenging. Besides, high-concentration grain boundaries and defects will easily form on as-grown TMDs during the CVD reaction, which does harm to the application of the electronic device. It has been widely acknowledged that the domain size of CVD-grown TMDs is associated with the nucleation density.^27^ The precursor concentration is an important factor to ensure the effective control of nucleus and epitaxial growth of TMDs during the CVD reaction period. In the early growth stage, the high density of metal-oxide vapor will result in high nucleation density. So it is necessary to ensure low density of the metal precursor concentration at the early growth stage.^39^ In this subsection, we will focus on the study of the following key factors of controlling nucleation density during the CVD reaction: (1) suitable substrate and (2) well-designed reaction systems.

**Fig. 3:**
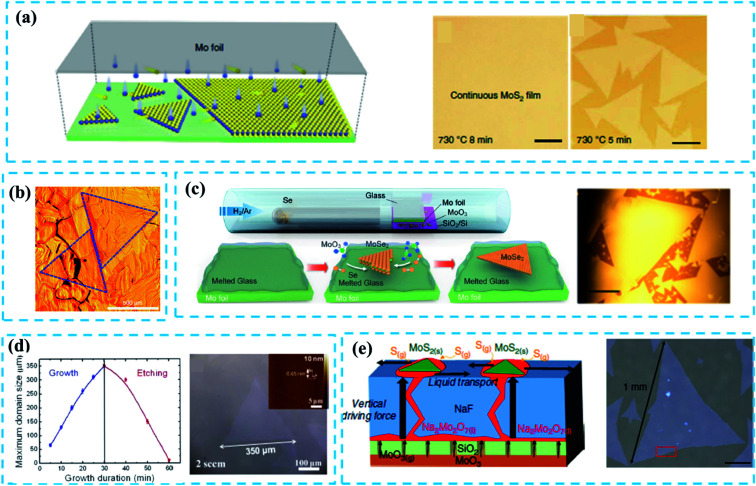
(a) Schematic diagram of a plane source supply system and typical optical images of MoS_2_ films grown on soda-lime glass by low pressure CVD (LPCVD), scale bar: 200 μm. Adapted with permission from ref. 45, copyright 2018 Nature. (b) Optical image of millimeter-size triangular monolayer WS_2_ single-crystalline domains on Au foil, scale bar: 500 μm. Adapted with permission from ref. 46, copyright 2017 Nature. (c) Schematic diagram (left) and optical images (right) of CVD growth of MoSe_2_ crystals on molten glass, scale bar: 500 μm. Adapted with permission from ref. 13, copyright 2017, American Chemical Society. (d) Evolution of the size of single-crystal MoS_2_ domains as a function of the growth duration (left); optical images of MoS_2_ (right) grown on sapphire after 30 min with O_2_ flow of 2 sccm by LPCVD, scale bar: 100 μm. Adapted with permission from ref. 38, copyright 2015 American Chemical Society. (e) Schematic diagrams of the growth mechanism (left) of the self-capping vapor–liquid–solid (SCVLS) method (right) and 1 mm MoS_2_ flake grown through the SCVLS method. Scale bar: 200 μm. Adapted with permission from ref. 32, copyright 2020 Nature.

As reviewed in ref. 47, it is necessary to select suitable substrates to effectively reduce the nucleation density and increase the domain size. Au-substrates are used widely to grow large-size TMDs because of their good catalytic surface.^4,14,46^ Typically, Gao *et al.*^46^ reported the CVD growth of a uniform WS_2_ monolayer film at the wafer-scale on the Au substrate that was composed of millimeter-scale single-crystalline domains as shown in Fig. 3b. The extremely low (below 0.1 atomic% at 800 °C) solubility of W in Au enabled the segregation and precipitation processes which suppressed the nucleation and multilayer growth of WS_2_ crystals; this was identified as the self-limited strategy (SLG) of the catalytic surface growth. In addition, the Au substrate can be reused in the growth of TMDs before a cleaning process.

Despite the advantages, the high price of gold foils and the complex cleaning process before reusing largely limit their applications.^46^ So, cheap and reliable substrates are demanded for the nucleation control of TMDs. Soda-lime glass^13,45,48–52^ with a smooth and highly uniform surface at high temperature, has found a wide usage in significantly suppressing nucleation.^13,47^ Besides, Na and Ca elements in the soda-lime glass structure can promote the growth of TMDs.^45^

Yang *et al.*^45^ reported a typical face source supply method based on the LPCVD system to fabricate 6-inch MoS_2_ films on a soda-lime glass with a domain size larger than 400 μm (Fig. 3a). The concentration of the S precursor was oversaturated due to the limited Mo evaporation, leading to a low nucleation density during the CVD growth process. Similarly, Loh *et al.*^13^ utilized a molten glass substrate to achieve the rapid growth of high-quality, millimeter-sized MoSe_2_ single crystals through an atmospheric-pressure CVD (APCVD) system (Fig. 3c). Quasi-atomic smooth and homogeneous liquid surface was generated when glass melted above 750 °C, which significantly reduced the nucleation density and thus promoted the growth of large-sized (up to 2.5 mm, Fig. 3c) MoSe_2_ crystals. The FETs based on as-fabricated MoSe_2_ show a carrier mobility of 95 cm^2^ V^−1^ s^−1^ and an on/off current ratio of 10^7^; this confirms the high quality of large-scale crystals. Besides, the soda-lime glass has a certain potential in controlling the layer number of TMDs.^49,52^ Although Na and Ca elements present in the soda-lime glass can promote the growth of the TMD film, these impurity elements may damage the quality of as-grown films.

Apart from the substrates, the modification of the reaction system can also play an important role in controlling the nucleation density. In 2015, Chen *et al.*^38^ reported triangular single-crystal MoS_2_ domains (with a maximum size of up to 350 μm in an optimal growth time: 30 min, Fig. 3d) that were synthesized on the sapphire substrate. By introducing a small amount of oxygen, the nucleation density is significantly reduced and the unstable nucleus is etched off. A similar case can be seen in ref. 53. Very recently, Chang *et al.*^32^ proposed a novel self-capping vapor–liquid–solid (SCVLS) method to obtain large single crystals and full-coverage MoS_2_ films with millimeter size. As depicted in Fig. 3e (left), the SiO_2_ layer acts as a diffusion membrane to control the diffusion of the MoO_3_ layer, and the NaF layer can react with MoO_3_ vapor that diffuses upward. Na_2_Mo_2_O_7_ is formed as an intermediate liquid phase through a eutectic reaction of MoO_3_ with NaF and, finally, is converted into MoS_2_ by sulfurization, wherein the MoS_2_ seeds are formed and act as a capping layer that ensures the low nucleation density and promotes lateral growth. However, the nucleation points are thicker than the epitaxial film, which inevitably reduces the uniformity of the whole sample.

Table 1 summarizes the recent progress in the fabrication of large-domain TMDs by controlled nucleation density, indicating the single-crystalline domain size of CVD-grown TMDs (up to 2.5 mm). However, the as-prepared single-crystalline TMD flakes can hardly meet the requirements of commercial purposes. First, the modern semiconductor industry prefers continuous uniform film at the wafer-scale, to ensure the stable performance and the convenient fabrication of devices. Second, the salt-assisted growth of TMD films inevitably induces the doping and the nonuniformity of products. Therefore, new approaches for the growth of TMDs with centimeter-scale single-crystalline domain size are still desired.

**Table 1:** A summary on the recent progress in the CVD growth of large-domain MX_2_ (M : Mo, W; X : S, Se)^a^

Materials	Domain size/μm	Substrate	Precursor	Growth *T*/°C	CVD	Carrier gas	Ref.
MoS_2_	170	*c*-Sapphire	MoO_3_, S	775	APCVD	N_2_	15
MoS_2_	350	*c*-Sapphire	MoO_3_, S	850	LPCVD	Ar/O_2_	38
MoS_2_	400	Soda-lime glass	Mo foil, S	730	LPCVD	Ar/O_2_	45
MoS_2_	500	Si/SiO_2_	MoO_3_, S	800	APCVD	Ar	19
MoS_2_	567	Soda-lime glass	MoO_3_, S	800	APCVD	Ar	51
MoS_2_	1000	NaF	MoO_3_, NaF	800	LPCVD	Ar/H_2_	32
WS_2_	430	Au foil	AMT, H_2_S	935	APCVD	N_2_/H_2_S	4
WS_2_	440	Si/SiO_2_	WO_3_, S	1100	LPCVD	Ar	16
WS_2_	1000	Si/SiO_2_	WO_3_, S	1300	APCVD	Ar	27
WSe_2_	800	Si/SiO_2_	WO_3_, S	1200	APCVD	Ar	27
WS_2_	1000	Au	WO_3_, S	800	APCVD	Ar	46
MoSe_2_	1600	Si/SiO_2_	MoO_3_, Se	750	APCVD	Ar/H_2_	24
MoSe_2_	2500	Soda-lime glass	Mo foil, MoO_3_, Se	1050	APCVD	Ar/H_2_	13

a AMT: ammonium metatungstate hydrate.

### 2.3 Orientated growth

As discussed, the fabrication of large-domain TMDs *via* nucleation control (Subsection 2.2) is performed at ∼mm level. Compared to the nucleation control, the orientated growth is a more beneficial strategy for the practice of fabricating large single-crystal TMDs.^54^ In fact, TMD films are usually composed of randomly-distributed aligned single crystals that merge with the adjacent flakes,^55^ along with numerous grain boundaries due to the mirror domains of TMDs which are always a problem. Therefore, it is challenging to achieve an orientated growth for TMD films with few grain boundaries overcoming the crystallographic limitation. In this subsection, we mainly focus on discussing the following two issues: the key factor for (1) controlling orientated growth and (2) eliminating the mirror domains of TMDs.

**Fig. 4:**
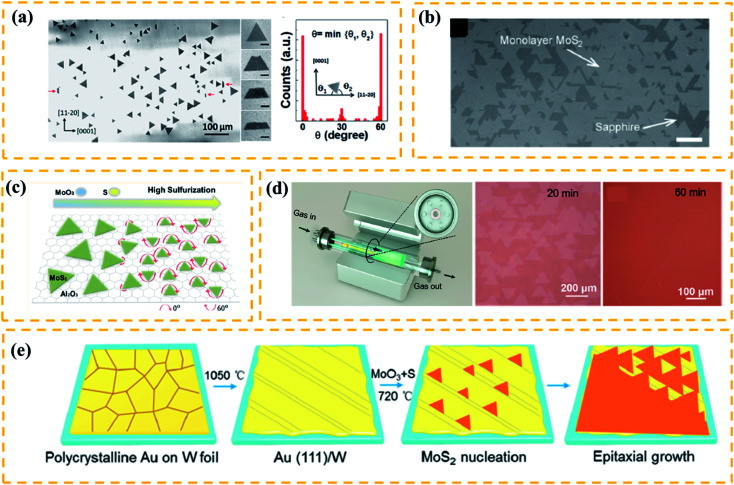
(a) Schematic diagram of a plane source supply system and typical optical images of MoS_2_ films grown on soda-lime glass by low pressure CVD (LPCVD), scale bar: 200 μm. Adapted with permission from ref. 45, copyright 2018 Nature. (b) Optical image of millimeter-size triangular monolayer WS_2_ single-crystalline domains on Au foil, scale bar: 500 μm. Adapted with permission from ref. 46, copyright 2017 Nature. (c) Schematic diagram (left) and optical images (right) of CVD growth of MoSe_2_ crystals on molten glass, scale bar: 500 μm. Adapted with permission from ref. 13, copyright 2017, American Chemical Society. (d) Evolution of the size of single-crystal MoS_2_ domains as a function of the growth duration (left); optical images of MoS_2_ (right) grown on sapphire after 30 min with O_2_ flow of 2 sccm by LPCVD, scale bar: 100 μm. Adapted with permission from ref. 38, copyright 2015 American Chemical Society. (e) Schematic diagrams of the growth mechanism (left) of the self-capping vapor–liquid–solid (SCVLS) method (right) and 1 mm MoS_2_ flake grown through the SCVLS method. Scale bar: 200 μm. Adapted with permission from ref. 32, copyright 2020 Nature.

It has been known that single-crystalline substrates, such as *c*-sapphire^55,60^ and GaN,^61^ can promote the aligned growth of TMDs due to their similar symmetry and compatible lattice constants. Researchers have also discovered that the substrates with different lattice symmetry from TMDs can drive an aligned growth. As shown in Fig. 4a, Wang *et al.*^56^ successfully prepared well-aligned WS_2_ film on an m-plane quartz substrate. Notably, this plane of quartz only has 2-fold symmetry and does not have a good lattice match with the TMDs. Similar results were seen in highly aligned MoS_2_ nanoribbons grown on a-plane sapphire (2-fold symmetric) substrate.^62^ It is believed that, due to the high temperature between the as-grown sample and the 2-fold symmetric substrate, the lattice mismatch induces anisotropic strains that regulate the aligned growth of crystal domains. Besides the substrate symmetry, the terraces have been considered as an important factor to guide the orientation growth.^55,57^ For example, Dumcenco *et al.*^57^ fabricated the aligned monolayer MoS_2_ on the *c*-sapphire substrate (Fig. 4b). Before reaction, the *c*-sapphire substrate was annealed at 1000 °C for 1 h to form long terraces and wide steps that promote the aligned growth of TMDs. Other reports, however, have shown an aligned growth of TMD triangles without pre-annealing.^63^ Therefore, the symmetry of the substrate and terraces/steps on the substrate should not play a key role in determining the aligned growth.

Many efforts have been made on the key factors that determine the orientation of TMDs. Ji *et al.*^64^ demonstrated aligned growth of monolayer WS_2_ on *c*-sapphire by introducing a high concentration of hydrogen during the CVD process. The experimental results and DFT calculations revealed that the high concentration of hydrogen can induce strong coupling between WS_2_ and the *c*-sapphire surface, facilitating an oriented growth. Besides, the precursor concentration during the CVD process has been shown to play an important role in modulating the orientation of nuclei.^54,56^ Aljarb *et al.*^58^ systematically studied the aligned growth of the MoS_2_ monolayer on *c*-plane sapphire. They found that the S : Mo ratio can effectively control the size and orientation of seeds, thus achieving well-orientated MoS_2_ monolayers. As shown in Fig. 4c, small seeds can be aligned easily at the early growth stage upon the high S : Mo ratio of the substrate, and in the next stage, the ratio should be decreased in order to increase the lateral size. Furthermore, Aljarb *et al.*^65^ also achieved an epitaxial growth of single-crystalline and oriented TMD nanoribbons *via* ledge-directed epitaxy (LDE)-assisted CVD. They found that the aligned nucleation of MoS_2_ seeds preferably took place at the ledges of β-Ga_2_O_3_(100), then the high S : Mo ratio environment facilitated the unidirectional growth of single-crystalline domains. Finally, these aligned single-crystalline domains merged into a continuous single-crystalline nanoribbon. Despite these findings, some fundamental parameters, such as pressure and temperature that may modulate the oriented growth of TMDs, have not been fully understood.

As discussed, satisfactory oriented TMD films should be composed of large single-crystalline flakes and few grain boundaries. Very recently, Wang *et al.*^59^ successfully prepared a highly oriented monolayer MoS_2_ film at a 4-inch scale on the *c*-sapphire substrate using a facile multisource LPCVD method. As shown in Fig. 4d(left), seven miniature quartz tubes in the growth chamber served as the pocket reaction sources, where the S source was loaded at the center of the mini-tube and MoO_3_ sources were evenly loaded in the six mini-tubes. This special multisource design ensured the homogeneous supply of precursors and precise control of reaction kinetics. As a result, the as-grown film had an average domain size of >180 μm (Fig. 4d). However, there are antiparallel domains (0 and 60° orientations) and inevitable mirror twin boundaries in most oriented TMD films, which could greatly degrade the performance of related devices.^66,67^

A recent theoretical study shows that the presence of atomic steps can break the equivalence of 0 and 60° domain orientations.^68^ Based on this idea, Yang^54^ reported the unidirectional nucleation-growth of wafer-scale single-crystalline MoS_2_ monolayers on vicinal Au(111) using an APCVD method. As depicted in Fig. 4e, the single-crystalline Au (111) film was obtained by melting and resolidifying commercial Au foils on W foils. By precisely controlling the S : Mo ratio during the reaction process, the as-prepared Au(111) surface ensured the synthesis of MoS_2_ films with an unidirectional alignment and seamless stitching. The DFT calculations revealed that the step edge of Au(111) could effectively suppress the nucleation of 60° oriented domains. This work successfully achieved the seamless splicing of large-scale MoS_2_ monolayers, however, the high price of substrates and the complicated processing procedures made it hard to utilize this technology for practical applications.

In summary, the oriented growth of TMDs needs a detailed modulation of precursor concentration to control reaction kinetic parameters, accompanied by a suitable substrate with terraces/symmetry. More fundamental details such as pressure and temperature about the oriented growth of TMDs need to be studied. Besides, a low-cost and simple method to eliminate the grain boundaries and improve the uniformity of aligned TMD films would be an in-demand topic for 2D structures in the near future.

## 3 Conclusions and outlook

In this review, we summarized recent developments on the controllable CVD growth of high-quality TMD films with large domain size, good uniformity, perfect crystal alignment, and few grain boundaries, which are highly desired for industrial production in the future.

It has been confirmed that the precise control of precursor vapor is the prerequisite for the controllable growth of TMDs films (as shown in Fig. 1), which can be achieved by the usage of appropriate precursors, well-designed reaction setups, specific experimental manifestations, *etc.* Besides, the nucleation control of TMDs during the CVD reaction is another important topic, affecting the domain size and the number of grain boundaries in as-grown films. The promoter effects of substrates have been confirmed to effectively increase the size of TMDs single crystals to millimeter scale. Specific substrates and modified reaction systems can also suppress the nucleation density of TMDs to obtain an increased domain size. It is concluded that single-crystalline substrates with atomic stages assisted by the fine control of reaction kinetics can promote the oriented growth of epitaxial TMDs films with limited grain boundaries at the wafer scale.

Despite the remarkable advances in the past years, the exploratory growth of satisfactory large-area and high-quality TMDs film is still in its infancy with many challenges faced:

(1) Because of the complicated evaporation process of the TM source, it is difficult to thoroughly eliminate the concentration gradient of the precursor in the CVD system, leading to uneven properties of as-grown films. Compared to the one-step fabrication route, the pre-deposition of TM precursors on the substrate before the reaction can be a useful method to obtain the uniform growth of TMDs films.

(2) As discussed above, soda-lime glass has been widely used as a low-cost substrate to increase the domain size of TMDs, wherein Na^+^ ions in the substrate are believed to promote the in-plane growth of TMDs, but the quality of as-grown films is decreased due to the inevitable chemical doping from them. Therefore, it is quite important to meticulously understand the promoter effect of Na^+^ ions in the growth of TMDs and develop an alternative strategy such as precise modulation of reaction kinetics to fabricate high-quality TMDs flakes with a large domain size without the impurity-doping.

(3) Recent advances have verified that the substrate with atomic stages can effectively decrease the antiparallel domains (0 and 60° orientations) in epitaxial TMDs films. Although sapphire or gold substrates can eliminate the twin boundaries in as-grown epitaxy TMDs films, the high price of substrates and complicated processing procedures for substrates make it hard in practice application. In addition, β-Ga_2_O_3_ substrates with atomic stages that can effectively promote the epitaxy growth of perfect TMDs films are very expensive and it is difficult to guarantee the quality of atomic stages. Therefore, economic substrates and efficient fabrication technologies of atomic stages are required for the epitaxy growth of wafer-scale TMD films.

## Author contributions

Qun Wang and Run Shi contributed equally to this review. The manuscript was finished with the contributions of all authors. All authors have given their approval to the final version of the manuscript.

## Conflicts of interest

There are no conflicts to declare.

## Supplementary Material
